# Review of Hmong-Related Health Problems: A Quick Guide for Healthcare Providers

**DOI:** 10.7759/cureus.9808

**Published:** 2020-08-17

**Authors:** Ali H Ali, Mandip S Kang, Kamalmeet Kaur, Saja Al adhami, Candice R Yuvienco

**Affiliations:** 1 Internal Medicine, University of California San Francisco-Fresno, Fresno, USA; 2 Internal Medicine, Community Regional Medical Center, Fresno, USA; 3 Internal Medicine/Rheumatology, University of California San Francisco-Fresno, Fresno, USA

**Keywords:** hmong, homng-related health issues, health disparities, epiddemiology

## Abstract

The people of Hmong descent are one of the largest resettled communities in the United States (US). The Central Valley of California is well known to be the home to the largest Hmong population in the US. However, despite the presence of such a large Hmong community in the Central Valley, our knowledge of their cultural perceptions of medicine is limited. Based on local Central Valley health providers’ experiences and observations, the Hmong people have a number of health-related challenges that differ from those of the general population, and this should be considered when dealing with their healthcare needs. In this report, we present a quick guide about the Hmong community and their health-related issues. We hope this will help clinicians and researchers better understand the Hmong community, which in turn would help provide a better quality of healthcare to the Hmong people and stimulate intellectual curiosity among healthcare providers towards this unique Asian ethnicity.

## Introduction and background

The Hmong are an Asian ethnic group originally from southern China that later migrated to the northern regions of Laos, Vietnam, and Thailand. Since the 1970s, large numbers of Hmong have come to the United States (US) as refugees with their last major resettlement happening in 2006 [1-2]. The aim of this review is to provide healthcare workers with a concise guide that should provide clarity regarding most of the Hmong-related health issues and their cultural beliefs that affect their management in order to enhance their care.

The Hmong in the US are predominantly from Laos and were recruited by the US Central Intelligence Agency to fight in the “Secret War” in Laos in 1965. Their role was to fight the communists in Laos, to prevent the North Vietnamese from coming through Laos into South Vietnam. The Secret War ended in 1975 with the conclusion of the Vietnam War. Many Hmong fled to refugee camps in Thailand and subsequently migrated to the US [2]. Eventually, the Hmong moved to California, Minnesota, Wisconsin, Ohio, and North Carolina, where large numbers of clan groups reside [3].

Having basic knowledge about Hmong traditions is an essential part of taking care of their healthcare needs. The Hmong clans play an important role in maintaining the mental and general well-being of the Hmong community. In the US, approximately 70% of the Hmong still practice traditional, animist ancestor worship and Shamanism, whereas the other 30% have converted to Christianity [4]. The shaman is an individual who, according to Shamanism, travels between the visible and the spirit worlds through ritual practices that are conducted for purposes of healing, divination, and control over natural events. They are believed to be important sources of mental health support for the Hmong community [5].

Healthcare providers should be mindful that the Hmong language lacks vocabularies and terms related to body anatomy, pathologies, and disease process. For example, there is no word in the Hmong language that refers to “cancer”. Therefore, interpreters may need to explain in more words and sentences to deliver the best description of the disease [6].

## Review

Hmong demographics

It is estimated that there are 309,564 Hmong in the US. California has the largest Hmong population followed by Minnesota and Wisconsin. The Hmong live mainly in the San Joaquin Valley area in California, and Fresno, Sacramento, and Merced are among the top 10 cities where they reside. Fresno has the second largest population of the Hmong in the US, after Saint Paul, Minnesota, which has the largest Hmong population in the US [7-8].

The Hmong people have a median age of 24.5 years compared to 38.1 years among the general population in the US; 60.6% of the Hmong are between the ages of 18 and 64, compared to 61.8% in the same age group among the general population. The Hmong who reside in the Central Valley of California mainly work in farming as they were influenced by the agricultural environment in the Valley. Similarly, in North Carolina, most of the Hmong run poultry and egg farms. On the other hand, many of the Hmong in Michigan have turned to owning and operating their own ventures within the food industry. Although the Hmong are in the process of developing their own distinct Hmong-American culture, they still preserve the best features of their language and traditions [8].

In terms of social determinants of health, current data suggest that the Hmong might be at risk of poor health conditions in general. The Hmong have an average household income of $45,776, compared to $66,201 among Asian Americans in general. Additionally, 23.2% of the Hmong have lower than a high school diploma compared to 12% of the general population, and 76.8% have higher than a high school diploma compared to 88% among the general population [7]. Also, 87% of Hmong in the US speak English less than very well, compared to 60% of the Asian American population in general [1].

Epidemiological research and survey data have improved our overall understanding of health disparities and how they arise, which in turn should help facilitate a better approach to community-based medical management. However, much of the research has focused on large (umbrella) racial and ethnic minority categories. For example, nationwide studies in the US, such as those conducted by the Centers for Disease Control and Prevention (CDC), National Center for Health Statistics (NCHS), and Behavioral Risk Factor Surveillance System (BRFSS) have defined the umbrella of racial and ethnic minorities as “White, Black or African American, American Indian or Alaskan Native, and Asian”, effectively making racial and ethnic minority subgroups invisible. This generalizes certain communities and aggregates them under one single group, irrespective of the vast differences within them. National health research has been conducted with Asian Americans as an aggregate group, or with the six larger Asian American subgroup populations (Asian Indian, Chinese, Filipino, Japanese, Korean, and Vietnamese) at a disaggregate level. The Hmong community, even with its diverse culture and beliefs, were accounted for under Asian Americans. Moreover, there are many other sizable Asian American groups in the US who, like the Hmong, have not been disaggregated in national studies: Pakistani (409,000); Cambodian (276,000); Thai (237,000); Laotian (232,000); Taiwanese (230,000); Bangladeshi (147,000); and Burmese (100,000). These groups have different cultural backgrounds and vastly different histories, including differences in diet, cultural practices, health-related beliefs, education, socioeconomic status, and geographical origins. Thus, “determinants of health,” such as income and education, may vary considerably between and among each of these subgroups and could result in significant differences in health outcomes. Little is known as to whether there are differences between the umbrella groups and any of the subgroups and within the subgroups themselves [1].

The Hmong’s conception about illnesses

According to Shamanism, the Hmong believe that diseases are caused by a departed ancestral spirit or the souls of those who have suffered an illness. The latter explains why certain Hmong believe that illness results from the separation of body and soul, which may or may not be related to the act of offending an ancestor, rather than a biological process. Moreover, the wish of the soul to leave the body it occupies is another concept related to the disease process according to the Hmong culture. As a result of these conceptions, the Hmong believe that western medicine is not the best treatment option for the illnesses, and they can instead be treated by a traditional healer called the shaman. The shaman is responsible for duties similar to that of a psychologist, a doctor, and a minister [5].

The shaman is the person who can ensure contact between the visible and the spirit world through specific spiritual activities. According to the Hmong culture, if a shaman’s ritual of soul-calling fails to bring back the soul to the sick individual, the person's condition might get worse, even leading to death [5,9]. Due to these beliefs, many Hmong families have difficulty in understanding the concept of disease transmission through microorganisms, and they may even refuse western medicine-based treatments [9]. Home remedies and herbal treatments are still widely used among the Hmong community. The importance of biomedicine is known among Hmong communities, but traditional diagnosis and herbal or spiritual treatments are usually accorded the first preference [5]. It is important for healthcare providers to be aware of these conflicts between biomedical practices and Hmong beliefs. For instance, Hmong people may be hesitant to undergo surgical procedures due to the belief that they may lose their soul while being unconscious under anesthesia. After any general anesthetic induction involving a Hmong patient, it may be important to conduct a soul-calling ceremony in the operating room to recall the lost soul. Furthermore, the surgical removal of a body part may clash with the Hmong belief in reincarnation, which explains why some Hmong refuse surgical interventions [5].

Hmong-related health issues

The Hmong community has been found to have higher rates of infection-related cancers like nasopharyngeal carcinoma, gastric cancer, hepatic cancer, and cervical cancer. The prevalence of these cancers among the Hmong is higher compared to the commonly found cancers among the rest of the US population, such as lung, breast, and colorectal cancers [10-11]. For instance, hepatitis B-linked liver cancer disproportionately affects Hmong Americans as the incidence rate is six times greater than that among non-Hispanic whites [12]. The Hmong are reported to have a high prevalence of hepatitis B virus, ranging from 3.41%-16.7%, which is on the higher side compared to the prevalence rate of 0.15%-1.27% with other Asians, Caucasians, African Americans, Hispanics, and Native Americans [13,14]. A low rate of Hepatitis B screening (24%) was reported among the Hmong adults in Sacramento [12]. Furthermore, Hmong has the highest liver cancer mortality rate compared to other Asian ethnicities [15]. Similarly, nasopharyngeal cancer mortality rates are 10.4 for the Hmong compared to 0.2 and 1.7/100,000 for non-Hispanic whites and Asians/Pacific Islanders, respectively [16]. Cervical cancer incidence and mortality rates among the Hmong women are three to four times higher than Asians/Pacific Islanders and non-Hispanic white women [17]. Due to poor access to health services, it is not surprising that the Hmong are more likely to be diagnosed with cancer at an advanced stage [15].

The main contributors to the high cancer morbidity and mortality among the Hmong are likely attributed to genetics, problems in access to health services, and diet-related issues. In a study of cancer-related genetic polymorphisms, the Hmong were found to have significant risk genetic factors associated with cancer etiology and prognosis [10,18-20]. A study in Minnesota showed that newly arrived Hmong refugees from Thailand had significant cardiovascular risk factors on arrival, including obesity, hyperglycemia, hyperuricemia, renal insufficiency, and hypertension [21].

Another study that investigated the clinical features of cerebrovascular accidents in the Hmong compared to non-Hispanic white people showed that the Hmong suffer from poorly controlled risk factors like diabetes and had higher incidences of small vessels and intracranial atherosclerotic disease, but low incidence of carotid disease and heart failure [22]. In Wisconsin, Thao et al. showed that the prevalence of diabetes among the Hmong was 11.3% compared to 6% in the non-Hispanic white population [23]. This is supported by another study that concluded that the Hmong are at a high risk of diabetes based on diabetes screening tools, especially the waist circumference and high-risk random blood glucose [24]. In the same context, a study in Minnesota showed that mean systolic and diastolic blood pressure in the Hmong children is higher than that in their white and African American counterparts, which puts them at higher risk of hypertension later in their lives [25].

Interestingly, several studies have been conducted to investigate gout and hyperuricemia among the Hmong after the discovery of the high prevalence of gout among them. It was found that the Hmong have a twofold higher prevalence of gout compared to the non-Hmong population in Minnesota [26]. Similarly, there is a fivefold higher incidence of ureteral and kidney stones among the Hmong compared to the non-Hmong [27]. Moreover, one study revealed that the Hmong had a 31.5% incidence of tophaceous gout compared to 10.7% among Caucasians [28]. This study identified a statistically higher prevalence of validated risk alleles in the US Hmong population compared with published data on people of European or Chinese ancestry. Specifically, six out of eight risk alleles were more prevalent in the Hmong than Europeans, and two out of three risk alleles were more prevalent in the Hmong than the Chinese [29]. This observation represents the genetic basis for the higher prevalence of hyperuricemia and gout in the Hmong. Historically, gout is known among the Hmong people as “Crazy Foot” or “Taw Vwm” [30].

Regarding chronic disease management, the Hmong are less likely to pursue treatment as compared to their fellow Asian American counterparts; and when they do, the disease process, such as cancer, is usually detected at a later stage, leading to an increased risk in mortality in this population. Particularly, two studies in California have shown a large discrepancy related to such health-seeking behaviors. One study reported that over 97% of the Hmong patients deferred treatment for nasopharyngeal cancer as compared to 25.6% of their Asian/Pacific Islander counterparts [1,16]. The second study documented that only 3% of the Hmong patients diagnosed with hepatocellular carcinoma received treatment in the form of local surgical treatment, resection, or liver transplantation when compared to 22% of Asian Americans who sought surgical treatment [1,15].

In one study, about 50% of 323 Hmong adults were found non-compliant with their prescribed hypertension medications [31]. Hmong remedies such as herbs, including plants and tree roots, have been preferentially used by the Hmong to treat diabetes [32]. It was also reported that Hmong markets in Minnesota sold medications that were not approved by the FDA [33]. Fortunately, a survey among the Hmong adults has shown that only 20% of the Hmong reported the daily use of tobacco, and about 40% reported ever using tobacco [34]. Additionally, another study has shown that the Hmong have the lowest rate of smoking among all Southeast Asian populations [35].

After working in the Central Valley and having direct contact with the Hmong population, it is evident that there is a disconnect between western medicine and their belief system in terms of dealing with their comorbidities and management plans. Our approach has generally been to utilize a virtual video conferencing interpreter or have an English-speaking family member in the room in the clinical setting. This helps to create a friendly and inviting atmosphere. We recommend outrightly acknowledging the language and culture barrier, ​as this helps to build trust and overcome the disparities. With any patient, it is only realistic to ask about their expectations for the visit and their future care. Members of the Hmong community are generally welcoming when it comes to discussions about medical management; however, they can be reluctant to make an immediate change. It has been our experience that frequent clinic visits help with patient-physician understanding and better overall care. Again, in the inpatient setting, we recommend discussing the disconnect between their culture and western medicine, asking for their expectations in terms of management, and allowing for either a virtual or actual live interpreter. We recommend stationing Hmong-speaking nurses to care for patients of Hmong culture, which would help build rapport. In the critical care scenario, it is important to recognize that a lot of the standard interventions such as central lines, transvenous pacers, arterial lines, and endotracheal tubes may not be a part of their belief system. Hence, constant discussions involving patients' families are necessary. We have observed that, during such discussions, the male members of the family are often primarily involved, and the eldest member is the decision-maker. Recognition of this fact may help with better timing of family meetings to accommodate the eldest male member, as most decisions will be made only with their consent. In our opinion, the biggest barrier to the management of Hmong patients is the failure to recognize their distinct culture and belief systems.

## Conclusions

There are many challenges that practicing providers face when treating people belonging to various ethnic cultures. Overcoming language barriers, poor medical literacy among the patients, their low socioeconomic status, and lack of compliance are just some of the challenges that need to be overcome; however, we believe that the biggest challenge is the ignorance about our patients and their cultural backgrounds. A good patient-provider relationship is of the utmost importance when conveying medical management, which can only be attained by recognizing the differences in their culture and respecting their values/beliefs. We hope that this guide will further minimize the barriers between healthcare providers and the Hmong community and help build that critical patient-provider relationship. We recommend more targeted studies about health-related perceptions and treatments among various ethnicities such as the unique Hmong population.
